# The Readiness Ruler as a measure of readiness to change poly-drug use in drug abusers

**DOI:** 10.1186/1477-7517-3-3

**Published:** 2006-01-25

**Authors:** Morten Hesse

**Affiliations:** 1 Centre for Alcohol and Drug Research, Aarhus University, Købmagergade 26E, 1150 Copenhagen K, Denmark

## Abstract

Readiness to change is a crucial issue in the treatment of substance use disorders. Experiences with methadone maintenance treatment (MMT) has shown that continuous drug and alcohol use with all its consequences characterize most MMT programs. In a prospective study of drug abusers seeking opiate agonist maintenance treatment in the City of Copenhagen, subjects were administered the Addiction Severity Index, and the Readiness Ruler for each of 11 different licit and illicit drugs by research technicians. Data was collected upon admission to the program and at a 18 month follow-up. Subjects who indicated they wanted to quit or cut down upon admission, reported less drug use at 18 month follow-up, after controlling for severity of drug problems at intake. Subjects who expressed readiness to change their drug use upon admission decreased their drug use. It is concluded that the Readiness Ruler measures a construct related to actual readiness, supporting its use in the clinical context.

## Introduction

Readiness and motivation to change addictive behaviours is an important issue in both our understanding of, and treatment for addictive behaviours. Previous research has shown predictive validity of measures of motivation, such as the University of Rhode Island Change Assessment Scale [1], or the Stages of Change Readiness and Treatment Eagerness Scale [2-4], although a few studies have failed to find predictive validity of measures of readiness to change [5,6]. Thus, one of the defining characteristics of a measure of motivation or readiness to change is that it predicts actual behaviour change, and this characteristic is indeed true of several measures that are widely used in research.

Most of these measures are based on the transtheoretical stages of change model [7], or measure readiness to engage in treatment [e.g., [8]].

Although the Readiness Ruler (RR), developed by Stephen Rollnick, has been used in training settings and is recommended for use in clinical settings, no scientific studies have been conducted of the RR. In fact, searching PUBMED gives only a single hit on the RR, namely a tutorial on how to work with addiction problems in a family physician setting [9].

## Method

As part of a follow-up study of enhanced or standard psychosocial treatment for opiate agonist maintenance patients, patients were interviewed after approximately two weeks of methadone or buprenorphine maintenance, and re-interviewed on average 18 months after treatment.

### Sample and procedure

The sample consisted of all consecutive intakes to opiate agonist maintenance treatment in the City of Copenhagen who agreed to participate in a study of enhanced psychosocial services for drug abusers between August of 2002 and October of 2003. Participants gave written consent to participate in the study, and were informed that the study was part of research and quality assurance in treatment. Interviews were conducted by research technicians under the author's supervision. It was stressed in interviews that personal information from the interview would not be shared with anyone outside the research institution, including the staff involved in their treatment.

Patients came either from a special clinic in Copenhagen providing enhanced psychosocial services to drug users, or one of the City's 4 treatment and rehabilitation centres, providing the standard psychosocial services.

### Instruments

Subjects were interviewed with the European Addiction Severity Index (EuropASI) [10], and the Readiness Ruler (RR). The EuropASI was administered at both baseline and follow-up, but the RR was administered only at baseline.

The EuropASI is the European version of the Addiction Severity Index [11], with some adjustments made to match the European context. The EuropASI measures current and past severity in 8 areas: medical, work (financial/support and work satisfaction), legal, social, family, drugs, alcohol, and psychiatric problems. For each of these areas, a composite score is calculated, representing the severity of problems in the past 30 days. Composite scores can range from 0 (indicating no problems) to 1 (indicating very serious problems). Two of these composite scores were the dependent variables of the current analysis, drugs composite score and alcohol composite score at follow-up.

In the RR, subjects are asked to rate for each drug ranging from 1 to 10, their degree of readiness to cut down or quit. Ratings can range from 1–10. Scores of 1–3 represent non-readiness to change, scores of 4–6 uncertainty, scores of 7–8 represent readiness, and 9–10 represent ongoing attempts at changing. Alternatively, respondents can answer "Don't use" for a drug that they do not use.

The original design was kept as it appears on the Motivational Interviewing homepage [12]. However, a few categories were changed to fit the target population (e.g., stimulants were sub-divided into amphetamine and cocaine, and steroids were dropped).

For the current study, the mean value of all those illegal drugs that were checked were used as a predictor variable.

For alcohol, subjects were grouped according to the following rule: If they responded "don't use" they were given a code of "not user". If they responded 1, they were given a code of "User, will not change". If they responded 2–10, they were given a code of "User, may consider change". The reason for this recoding of the alcohol scale is that while all subjects were current drug users, many were not alcohol users, and thus estimating a degree of readiness for alcohol users would result in a much-reduced sample size.

### Statistical analysis

Multiple regression was used to assess the impact of readiness to change at admission on outcome at 18 months follow-up, using the drug use composite score at follow-up as the dependent variable, and controlling for baseline drug use composite score. Statistical analysis was carried out on STATISTICA 6.1 for Windows [13]. In the case of alcohol, the RR codes were entered as a categorical variable in the regression model with the values "Not user", "User, will not change", "User, may consider change".

## Results

### Sample description

A total of 87 subjects were entered in this part of the study. Subjects were consecutive admissions to the City of Copenhagen for maintenance treatment with either methadone or buprenorphine. A total of 67 subjects were re-interviewed after 508 days (range: 227–814), but due to missing data, this was reduced to 55 subjects for the present analyses. Comparisons of these 55 subjects to the 32 subjects with incomplete data revealed few differences on any intake variables. Those lost to follow-up had used less money on drugs than interviewed subjects (p = 0.04), and had less medical problems at intake (p = 0.02). There were no significant differences on gender or age, on the RR, or on any other EuropASI variable. The main factor in predicting attrition from the study was the setting, as most of the drop-outs came from the 4 standard treatment centres. The mean age at intake was 36.0 (SD = 8.4), and the sex distribution in the sample was 74% male and 26% female.

The descriptive characteristics of the RR and the EuropASI are summarized in table 1. Mean values of RR scales apply only to the percentage who used a given drug. The most commonly used drugs were tobacco, methadone, heroin and cannabis. Cocaine was also fairly common, reported by more than half the sample. Readiness to change was highest for heroin and illicit opiates, except methadone, followed by amphetamine and cocaine. The average subject was reluctant to make a change with regard to alcohol, cannabis or methadone, and not ready to consider quitting smoking tobacco.

**Table 1:** Descriptive statistics

	**%**	**Mean of those reporting use**	**Std. dev.**
**Readiness ruler values**			
Alcohol	53%	3.6	3.6
Cannabis	69%	3.9	3.3
Benzodiazepines	37%	5.5	3.5
Cocaine	59%	6.7	3.5
Amphetamine	13%	7.6	3.7
Heroin	79%	8.2	2.4
Methadone	86%	3.7	3.4
Other opiates	11%	7.8	3.3
"Party" drugs (e.g., MDMA)	8%	6.4	5.1
Tobacco	95%	1.7	1.9
Proportion of illicit drugs in which change is considered		70%	30%
**EuropASI-scores**		**Mean of all subjects**	**Std. dev.**
Medical		0.31	0.39
Financial		0.92	0.20
Work		0.18	0.34
Alcohol		0.10	0.18
Drugs		0.35	0.12
Legal		0.25	0.33
Family		0.16	0.29
Interpersonal		0.30	0.32
Psychiatric		0.17	0.22

**Notes**: N for the mean values of the RR varies, since subjects who did not report current use of a substance did not report readiness to change.

The large majority of the sample were unemployed as reflected in the very high employment CS. In other areas, subjects were slightly less severe than the average from a recent German study of opiate users [14].

### Readiness and substance use outcome

I tested whether baseline readiness to change predicted drug/alcohol use at follow-up through multiple regression analyses.

It was found that the RR was significantly related to drug use and alcohol use at follow-up. The results of the regression analysis are summarized in table 2.

**Figure 1:**
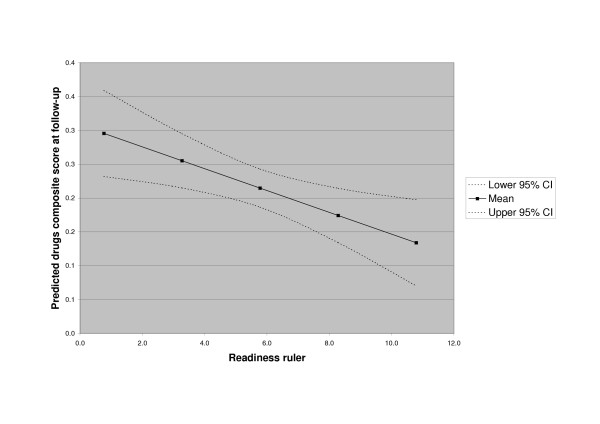
Predicted drugs composite score by Readiness Ruler score.

The mean RR score for all illicit drugs was significantly related to reduced drug use at follow-up after controlling for baseline value (beta = -0.35, p = 0.006), although baseline drug use was also significantly related to drug use severity at follow-up (beta = 0.24, p = 0.04). The relationship is shown in figure 1.

**Figure 2:**
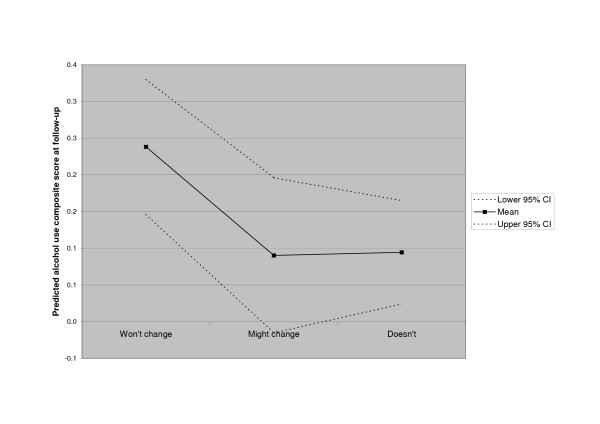
Predicted alcohol composite score by Readiness Ruler alcohol category.

Baseline alcohol use was strongly associated with alcohol use at follow-up (beta = 0.47, p = 0.0002). However, readiness to change drinking was also significantly related to drinking at follow-up. Subjects reporting "User, may consider change" did not report more or less alcohol at follow-up than those reporting being non-users at intake (beta = -0.18, p = 0.2). Subjects reporting "User, won't change" reported more alcohol use at follow-up than either of the two other groups, controlling for baseline use (beta = 0.38, p = 0.01). The relationship is illustrated in figure 2.

However, the possibility was considered that "readiness" or motivation could simply be a global feature, irrespective of type of drug. The analyses were therefore repeated, but this time using the RR illicit drug score to predict alcohol use at follow-up, and the alcohol readiness grouping to predict drug use at follow-up. None of these relationships were significant, consistent with the hypothesis that it is the specific readiness to change drug use, rather than global motivation that predicts behaviour change.

## Discussion

The current study added further support to established findings: that a person's expression of readiness to change behaviour is related to actual change. The RR, although a very simple measure of change readiness, could actually be used to examine readiness to change in poly-drug abusers entering methadone or buprenorphine agonist maintenance treatment.

From a clinical point of view, the RR has the advantage over many questionnaires used to measure readiness to change that it is very simple and face valid, and asks questions that are highly relevant in a clinical setting. Furthermore, it covers a range of different drugs, thereby ensuring that the clinician can identify and discuss the different drugs that the patient may use.

## Conclusion

The readiness ruler as an instrument measures a construct that is related to actual behavioural change. Low scores on the various rating scales generally was predictive of an absence of change, both specifically for alcohol and generally for drugs.
